# Dissecting the Gene Expression Networks Associated with Variations in the Major Components of the Fatty Acid *Semimembranosus* Muscle Profile in Large White Heavy Pigs

**DOI:** 10.3390/ani11030628

**Published:** 2021-02-27

**Authors:** Martina Zappaterra, Silvia Gioiosa, Giovanni Chillemi, Paolo Zambonelli, Roberta Davoli

**Affiliations:** 1 Department of Agricultural and Food Sciences (DISTAL), University of Bologna, Viale Fanin 46, I-40127 Bologna, Italy; paolo.zambonelli@unibo.it; 2 CINECA SuperComputing Applications and Innovation Department (SCAI), Via dei Tizii 6, I-00185 Roma, Italy; s.gioiosa@cineca.it; 3 Dipartimento per la Innovazione nei sistemi Biologici, Agroalimentari e Forestali (DIBAF), La Tuscia University of Viterbo, Via S. Camillo de Lellis, I-01100 Viterbo, Italy; gchillemi@unitus.it

**Keywords:** muscle acidic composition, fat, meat, pork, swine, mRNA, co-expression network, WGCNA, RNA sequencing, intramuscular fat

## Abstract

**Simple Summary:**

The amount and fatty acid composition of intramuscular fat are important features for the qualitative characteristics of processed and fresh meat products, but the knowledge of the key molecular drivers controlling these traits is still scant. To this aim, the present study investigated the co-expression networks of genes related to variations in the major fatty acids deposited in pig *Semimembranosus* muscle. Palmitic and palmitoleic acid contents were associated with a downregulation of genes involved in autophagy, mitochondrial fusion, and mitochondrial activity, suggesting that the deposition of these fatty acids may be enhanced in muscles with a reduced mitochondrial function. A higher proportion of oleic acid and a reduction in the percentages of *n*-6 and *n*-3 polyunsaturated fatty acids were related to changes in the mRNA levels of genes involved in Mitogen-Activated Protein Kinase (MAPK) signaling. The obtained results indicated gene expression networks and new candidate genes associated with the studied traits. Further studies are needed to confirm our results and identify in the discussed genes molecular markers for future selection schemes aimed at improving pork nutritional and technological quality. Furthermore, as pigs are considered reliable animal models for several human conditions, the obtained results may also be of interest for improving the knowledge of the molecular pathways associated with obesity and diabetes.

**Abstract:**

To date, high-throughput technology such as RNA-sequencing has been successfully applied in livestock sciences to investigate molecular networks involved in complex traits, such as meat quality. Pork quality depends on several organoleptic, technological, and nutritional characteristics, and it is also influenced by the fatty acid (FA) composition of intramuscular fat (IMF). To explore the molecular networks associated with different IMF FA compositions, the *Semimembranosus* muscle (SM) from two groups of Italian Large White (ILW) heavy pigs divergent for SM IMF content was investigated using transcriptome analysis. After alignment and normalization, the obtained gene counts were used to perform the Weighted Correlation Network Analysis (WGCNA package in R environment). Palmitic and palmitoleic contents showed association with the same gene modules, comprising genes significantly enriched in autophagy, mitochondrial fusion, and mitochondrial activity. Among the key genes related to these FAs, we found *TEAD4*, a gene regulating mitochondrial activity that seems to be a promising candidate for further studies. On the other hand, the genes comprised in the modules associated with the IMF contents of oleic, *n*-6, and *n*-3 polyunsaturated FAs (PUFAs) were significantly enriched in Mitogen-Activated Protein Kinase (MAPK) signaling, in agreement with previous studies suggesting that several MAPK players may have a primary role in regulating lipid deposition. These results give an insight into the molecular cascade associated with different IMF FA composition in ILW heavy pigs. Further studies are needed to validate the results and confirm whether some of the identified key genes may be effective candidates for pork quality.

## 1. Introduction

Global meat demand is estimated to be 16% higher in 2025 than in the 2013–2015 period, with poultry and pork representing the most consistent meat production and demand increase in developing countries [1]. Italy is among the main producers of processed meats, contributing about one-third of the European charcuterie [2] and being the first producer in Europe for Protected Designation of Origin (PDO) certified meat products [2]. The contribution of Italy to PDO production is relevant, particularly for high-quality dry-cured hams (such as Parma and San Daniele PDO hams) [3]. These products are obtained from hind legs from heavy pigs, which are specifically selected to fit the objectives required for these products [4,5], including slaughter at a minimum age of nine months and about 150–170 kg live weight. The genetic types used for heavy pig production show specific genetic and phenotypic features; carcasses and cuts have a balanced deposition of lean mass and fat, which make these meats suitable for processing.

The amount and fatty acid (FA) composition of subcutaneous and intramuscular fat (IMF) is a significant feature for the qualitative characteristics of processed and fresh meat products. Indeed, dry-cured ham quality greatly depends on thigh covering fat and IMF deposition, as an appropriate fat layer and higher amount of IMF are required for improved organoleptic characteristics and have been proven to prevent hams from experiencing excessive processing losses [4]. The amount and composition of IMF are of great importance for the organoleptic and nutritional quality of fresh meat, affecting also consumers’ perception and acceptance of pork products [6]. Even though an increase in the degree of lipid unsaturation in meat could be beneficial for human health [7,8], polyunsaturated FAs (PUFAs) are more likely to incur oxidative phenomena [9], making these FAs undesirable for the processing industry. Therefore, the pork processing industry’s technological requirements and consumers’ dietary demands do not completely match. These contrasting requirements create the necessity to find a balance between these divergent needs. So far, efforts have been made to improve both technological and nutritional quality traits, and an extensive body of literature focuses on the multiple variation factors affecting meat quality and FA composition of the final meat products [5,6,9,10]. Studies carried out in Italian Large White (ILW) pigs found that the FA composition of IMF can be improved through selection [11] and is associated with genetic markers [12]. In fact, muscle FA composition showed low to medium heritability estimates, ranging from 0.157 to 0.237 in ILW pigs. In particular, *n*-6 FA showed positive genetic correlations with carcass lean mass, while being negatively related to backfat thickness, suggesting that FA deposition in muscle is also related to carcass composition [13]. However, the exact molecular basis of muscle FA composition is still relatively unknown.

In the last ten years, high-throughput sequencing technologies have been used for transcriptome analysis, which has allowed the exploration of the gene co-expression networks in an unprecedented manner in terms of accuracy and data insight [14]. To date, the molecular networks associated with muscle FA composition have been investigated in several pig populations [15,16,17], but no information exists about gene co-expression networks related to FA composition in ILW heavy pigs. In Landrace x Iberian crossbred pigs slaughtered at about six months of age, individuals with high PUFA deposition in the *Longissimus dorsi* muscle were characterized by transcriptomic profiles, indicating an inhibition of glucose uptake and lipogenesis, which would produce a decrease in the muscle triglyceride storage [15]. In a sample of White Duroc × Erhualian pigs, muscle and liver FA composition was shown to be associated with genes involved in insulin resistance and in the immune system [16]. The latter result was in agreement with the literature, where adipogenesis has been indicated in humans and pigs to be implicated in inflammatory responses [18,19,20], suggesting that studying fat traits in pigs may also provide insights for a better comprehension of human disease genetic background.

In humans, several pathological conditions, such as obesity and diabetes, have been associated with changes in the FA composition of muscle and adipose tissue of the patients [21,22,23,24]. In humans, low contents of long-chain PUFAs and high amounts of palmitic acid in muscle were found to be linked with obesity [24]. On the other hand, high contents of *n*-3 and *n*-6 PUFA in adipose tissue were related to smaller adipocyte size [23]. Thus, as suggested by Zhang et al. [16], reaching a better understanding of the gene networks associated with FA composition in porcine tissues could provide information concerning lipid metabolism that may be of interest also for a better understanding of obesity-related pathological conditions in humans.

Therefore, shedding light on the genetic factors underlying muscle FA composition may be of paramount importance for the pig production industry and may also provide useful information for other species. Our previous study reported the gene expression networks associated with *Semimembranosus* muscle (SM) IMF deposition and the genes differentially expressed in two groups of ILW heavy pigs divergent for SM IMF content [13]. The current study starts from the results described in our previous research [13] and aims at investigating the co-expression networks of genes related to the variability noticed for the major FAs (palmitic, palmitoleic, stearic, and oleic acids) and FA classes (*n*-6 and *n*-3 PUFAs) in pig SM. These results allow for the identification of genes associated with FA composition and suggest possible genetic markers for the improvement of meat nutritional and technological quality.

## 2. Materials and Methods

### 2.1. Sampling and Phenotypes

The present research was conducted on the same set of samples already used in our previous paper [13]. This set of samples consisted of 12 individuals, chosen from a purebred population of 949 sib-tested ILW pigs, for their extreme and divergent contents of SM IMF (low IMF vs. high IMF group). A detailed description of the population of origin and of the 12 samples was reported in Zappaterra et al. [11] and Zappaterra et al. [13], respectively. The pigs entered the testing station at about 30 kg live weight and were reared in the same environmental conditions. During the testing period, pigs were single-stabled and fed the same finishing diet at a *quasi ad libitum* feeding level (i.e., about 60% of pigs were able to ingest the entire supplied ration). The sib test ended when the pigs reached an average final live weight of about 150 kg at about eight months of age. Animals were then transported to a commercial slaughterhouse according to Council Rule (EC) No. 1/2005 on the protection of animals during transport and related operations. The 949 pigs were slaughtered on 27 different days; after being electrically stunned, the animals were bled in a lying position in agreement with Council Regulation (EC) No. 1099/2009 on the protection of animals at the time of the slaughtering. After slaughter, SM samples were collected from the same carcass side of the 949 ILW pigs. The SM samples were then immediately frozen in liquid nitrogen and stored at −80 °C in a deep freezer until further analysis. As previously described [25], IMF content was determined for all gathered samples by extraction with petroleum ether from 1 g of SM using an XT15 Ankom apparatus (Ankom, Macedon, NY, USA), according to Official Procedure AOCS Am 5-04 [26]. IMF was determined in % (g/100 g of muscle tissue). SM FA composition was determined as described in Zappaterra et al. [11]. The FA and FA classes were expressed as mg/g of IMF. To avoid as much as possible other confounding effects, the two groups of samples (low and high IMF groups) were obtained balancing for sex and avoiding full and half-sibs. The 12 samples were slaughtered on nine different days.

### 2.2. RNA Extraction, Library Preparation, and Sequencing

A detailed description of the RNA extraction, library preparation, and sequencing is reported in Zappaterra et al. [13]. After total RNA extraction and quality checks were performed, stranded total RNA libraries were prepared, converting RNA to cDNA and adding sequencing adapters. RNA libraries were analyzed with a paired-end sequencing strategy on Illumina HiSeq2500 (Illumina Inc., San Diego, CA, USA). This strategy consisted of extracting, through ultra-high-throughput sequencing, short reads from both ends of cDNA fragments. The libraries were tagged, and pairs of libraries were run on a single lane. Paired-end reads of 100 bp were generated. The obtained raw sequence data have been deposited in the Gene Expression Omnibus (GEO) expression database under the accession number GSE144780.

### 2.3. Mapping, Assembly of the Reads, and Production of the Gene Count Matrix

Raw reads were obtained in FASTQ format and were quality-assessed using the FastQC program (retrieved from URL: http://www.bioinformatics.babraham.ac.uk/projects/fastqc/, accessed on 18 March 2020). As reported in Zappaterra et al. [13], the quality of the raw reads was measured according to sequence-read lengths and base-coverage, nucleotide contributions and base ambiguities, and quality scores and over-represented sequences. Trimmomatic utility [27] was used to trim out terminal low-quality bases and adaptor sequences, according to the FastQC reports. In particular, sequences with a quality below 15 in a sliding window of 4 consecutive bases were filtered out, and the remaining reads were retained if they had at least a 36-nucleotide length. The splice-aware read mapper HiSat2 [28] was used to align the clean reads against Ensembl reference genome *Sus scrofa* v. 11.1 (retrieved from URL: http://igenomes.illumina.com.s3-website-us-east-1.amazonaws.com/Sus_scrofa/Ensembl/Sscrofa11.1/Sus_scrofa_Ensembl_Sscrofa11.1.tar.gz, accessed on 18 March 2020). After alignment, one BAM file was obtained for each couple of fastq files. BAM files were further processed with Stringtie [29], with the aim of assembling the sequences into known transcripts. HTSeq version 0.6.1 [30] was then used to quantify the reads and obtain the file with the gene counts. The raw gene counts were then normalized with regularized-logarithm transformation (i.e., rlog) using the DESeq2 package [31] in the R environment [32]. The normalized gene counts were used to perform a Principal Component Analysis (PCA) in the R environment [32] to evaluate the dataset multivariate structure.

### 2.4. Weighted Gene Correlation Network Analysis (WGCNA)

The genes whose expression was not detected in most of the samples or with variance zero were filtered out from the normalized gene count matrix. To identify strong co-expression networks of genes related to SM FA composition, we employed a co-expression analysis approach using the “WGCNA” package [33] in the R environment [32]. The gene count matrix was analyzed together with the IMF contents of palmitic, palmitoleic, stearic, oleic, *n*-6, and *n*-3 FAs. These FAs were chosen for their relative abundance in IMF (palmitic, palmitoleic, stearic, and oleic are the saturated FAs (SFAs) and monounsaturated FAs (MUFAs) most represented in pork fat tissues [6,11]) and for their nutritional value (*n*-6 and *n*-3 PUFAs).

To obtain the scale-free undirected co-expression networks between the genes in the gene count matrix, an adjacency matrix was built using Pearson’s correlations between each gene couple. The soft threshold power values were estimated through the function *pickSoftThreshold()* in the WGCNA package, and a soft threshold power (β) of 6 was used to raise the adjacency matrix. This value was chosen as the scale-free topology index (R^2^) reached the peak (R^2^ > 0.70) for the first time when β = 6, and the minimum module size was 30 genes (Supplementary Figure S1). After β was determined, the adjacency matrix was calculated using the topological overlap measure (TOM) and the corresponding dissimilarity (dissTOM = 1 − TOM). The latter was used as a distance for gene hierarchical cluster, and then DynamicTree Cut algorithm [33] was used to identify the modules of genes. The default minimum cluster merge height of 0.25 was retained. After the construction of the gene co-expression network, WGCNA restituted a list of gene modules, which were named using color labels and clustered highly interconnected genes. The principal component of each module was defined as the module eigengene (ME); MEs represented the expression value of each module and were used to detect modules that may comprise genes having a biologically relevant role in the variations of the traits. The module-trait relationship (module membership, MM) was calculated as the Pearson’s correlation between the ME and the traits of interest. For each gene, the gene MM and the relative *p*-values were obtained and carefully evaluated, as they indicated the importance of that gene in a module. Due to the high number of gene modules showing a significant correlation with the analyzed traits, we decided to choose the gene modules for further analysis based on the threshold reported by Pampouille et al. [34]. Based on this study, only the gene modules with an absolute value of module-trait correlation higher than 0.7 were further analyzed. After selecting the gene modules exceeding the set threshold, the genes participating in those modules with a MM *p*-value < 0.001 were further considered to perform the functional enrichment analysis. The intramodular connectivity and gene significance (i.e., a coefficient and relative significance value indicating the importance of that gene for the trait, based on the correlation between the gene expression profile and the trait) were used to identify key genes in the networks.

### 2.5. Functional Enrichment Analysis of the Genes in the Significant Modules

To explore the biological functions of the genes significantly entering (MM *p*-value < 0.001) the identified gene modules (module-trait correlation higher than |0.7|), we performed Gene Ontology (GO) term enrichment analysis, as well as pathway ontology analyses using the Database for Annotation, Visualization and Integrated Discovery (DAVID) 6.8 online tool [35] and Cytoscape version 3.8.2 [36]. Cytoscape functional enrichment analysis was obtained using ClueGO [37] and CluePedia [38] plug-ins. *Homo sapiens* was used as the reference organism for both DAVID and Cytoscape functional enrichment analyses. Bonferroni adjusted *p*-values < 0.05 were considered significant for DAVID results, and Bonferroni step-down adjusted *p*-values < 0.05 were considered significant for Cytoscape functional enrichment analysis. For a better representation of the Cytoscape results, we applied the GO term fusion, in order to reduce redundant terms [37].

## 3. Results

### 3.1. Descriptive Statistics

The original ILW pig population (949 pigs) described in our previous paper [11] had an average content of IMF of 2.15 ± 1.13% and a hot carcass weight of 114.50 ± 8.64 kg. In that population, the SM FAs most represented were palmitic acid (with an average content of 23.51 ± 1.14%), palmitoleic acid (2.93 ± 0.49%), stearic acid (11.88 ± 1.19%), oleic acid (40.72 ± 2.89%), *n*-6 PUFAs (13.96 ± 3.12%) and *n*-3 PUFAs (0.60 ± 0.12%). Table 1 reports the sex, carcass weight, SM IMF content, and SM FA composition for the 12 samples selected from the 949 pigs and considered in the present study.

### 3.2. Mapping Results and Weighted Gene Co-Expression Network Analysis

As we detailed in our previous study [13], a total of 3,235,579,132 paired-end reads were obtained from the SM transcriptome sequencing of the 12 samples. After trimming and filtering steps, 1,155,025,434 reads were retained and mapped against the *Sus scrofa* reference genome 11.1. The total % of mapped reads (unique and multi-reads) was between 90% and 94%, and the uniquely mapped reads ranged between 79.1% and 85.7% across samples (Supplementary Table S1). The mapped reads were then assembled into transcripts, and the normalized gene counts were used to assess the multivariate structure of the dataset with a PCA. The results of the PCA showed no clear clusterization between samples (Supplementary Figure S2). 

**Figure 1:**
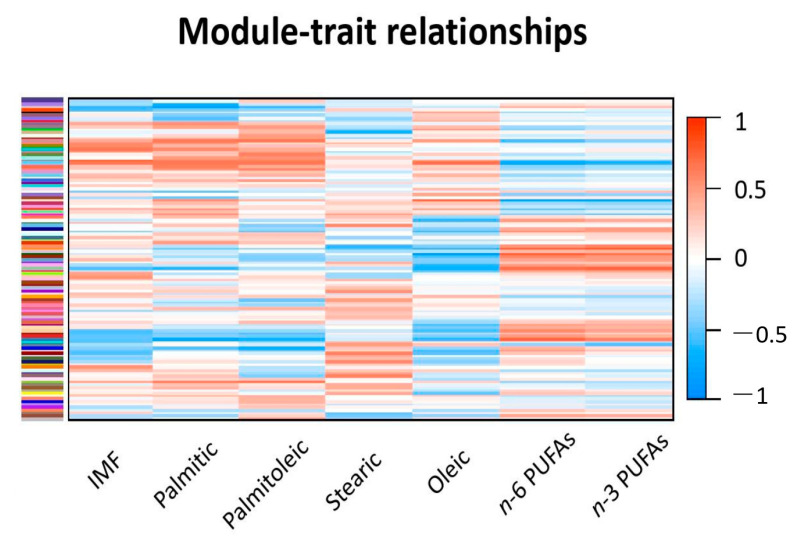
The heatmap graphically displaying the correlations between the gene modules and the studied traits. The scale bar on the left shows the strength of the correlations: the darker the color, the stronger the correlation. Correlations in light colors are of small magnitude and not significant.

The gene count matrix with the rlog values was then submitted for weighted gene co-expression analysis with the WGCNA package. After filtering out 5554 genes with missing expression values in most of the samples or with zero variance, a total of 20,046 genes were used for weighted gene co-expression network construction. As a result of network construction, 113 modules (i.e., cluster of co-expressed genes) were found by WGCNA analysis. These modules were identified by different color names, and their size ranged from 3374 to 30 genes with ME *p*-value < 0.05. The correlations between the 113 gene modules and the SM traits are displayed with a heatmap in Figure 1. The heatmap shows the strength of the correlations between modules and traits: the darker (dark blue and dark red) the colors, the stronger the correlations. As can be noticed, palmitic and palmitoleic acids show similar patterns of colors (and thus similar correlations with the modules) when compared to IMF. Oleic acid shows opposite correlations when compared to *n*-3 and *n*-6 FA. In fact, the modules that were positively correlated with oleic acid were negatively related to the percentage of *n*-3 and *n*-6, and vice versa. On the other side, stearic acid showed patterns of correlations different from the ones noticed for the other traits and of weaker magnitude, as indicated by the lighter colors found in the stearic acid column.

The complete list of the module-trait relationship values and the relative significances are reported in Supplementary File S1. The strongest and most significant module-trait correlations are reported in Table 2. The modules grey60, skyblue1, and lavenderblush3 were already discussed in our previous study on IMF deposition [13]. These modules showed significant module-trait correlations also with some of the FAs considered in the present study, but additional modules were identified. Palmitic acid was negatively correlated with lavenderblush3 (r = −0.80; *p*-value = 0.002) and with steelblue (r = −0.80; *p*-value = 0.002). The same two modules were also correlated with palmitoleic, but with correlation coefficients of lower magnitude (r = −0.60 and −0.65; *p*-values = 0.038 and 0.022, respectively). Palmitoleic acid showed only one module (yellowgreen, r = 0.71; *p*-value = 0.009) above the threshold of |0.70|. Oleic, *n*-6, and *n*-3 FA were correlated with the same modules (i.e., skyblue1, antiquewhite2, darkorange, darkseagreen3, darkgreen, and steelblue). In some cases, these modules had module-trait correlation values ≥ |0.70| for all three traits (i.e., antiquewhite2, darkorange, darkgreen); in other cases, the modules had module-trait correlation values ≥ |0.70| for some FAs among oleic, *n*-6, and *n*-3 PUFAs but were significant (*p*-value < 0.05) also for all of them (i.e., skyblue1, darkseagreen3, and steelblue). Stearic acid did not show module-trait correlations above the set threshold.

As can be noticed from Table 2, the considered FAs were clustered into two groups: on one hand, palmitic and palmitoleic acids were associated with the same gene modules; on the other hand, oleic acid was associated with the same gene modules found for *n*-3 and *n*-6 PUFAs, but with opposite correlation coefficients. That is to say, genes whose expression was positively correlated with *n*-6 and *n*-3 PUFAs (such as those included in the darkorange and darkgreen modules) were negatively correlated with oleic acid deposition, and vice versa. For these reasons, two functional analyses were performed: one for the genes included in the modules mostly associated with palmitic and palmitoleic, and the other for the genes included in the modules related to oleic acid, *n*-3, and *n*-6 PUFAs.

The complete list of the gene MM and the relative *p*-values (indicating the importance of each gene in the identified modules) are reported in Supplementary File S2. The overall gene significance of each gene on the studied traits is reported in Supplementary File S3.

### 3.3. Functional Enrichment Analyses

**Figure 2:**
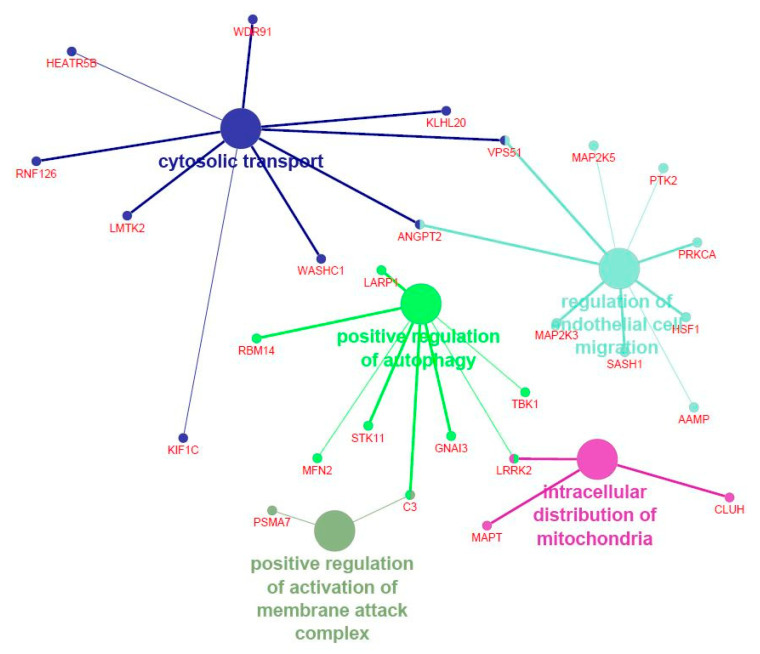
Functional enrichment analysis of the genes included in the modules significantly associated with palmitic and palmitoleic acids in pig *Semimembranosus* muscle. Different colors are used to highlight the significant Gene Ontology (GO) terms and the main genes entering in each GO term.

The genes significantly entering in the modules most correlated with the FA composition were then submitted for functional analysis. Supplementary File S4 reports the functional categories identified with DAVID for the genes significantly entering in the modules associated with palmitic and palmitoleic acids (i.e., yellowgreen, lavenderblush3, and steelblue). Figure 2 shows the Cytoscape functional annotation enrichment for the same genes. The genes included in yellowgreen, lavenderblush3, and steelblue modules were significantly enriched in “GO:0016482 Cytosolic transport” (*p* = 0.01), “GO:0048312 Intracellular distribution of mitochondria” (*p* = 0.02), “GO:0001970 Positive regulation of activation of membrane attack complex” (*p* = 0.02), “GO:0010508 Positive regulation of autophagy” (*p* = 0.02), and “GO:0010594 Regulation of endothelial cell migration” (*p* = 0.04). On the whole, both functional analysis tools suggested that the genes included in yellowgreen, lavenderblush3, and steelblue modules were significantly enriched in pathways controlling the organization of intracellular organelles.

**Figure 3:**
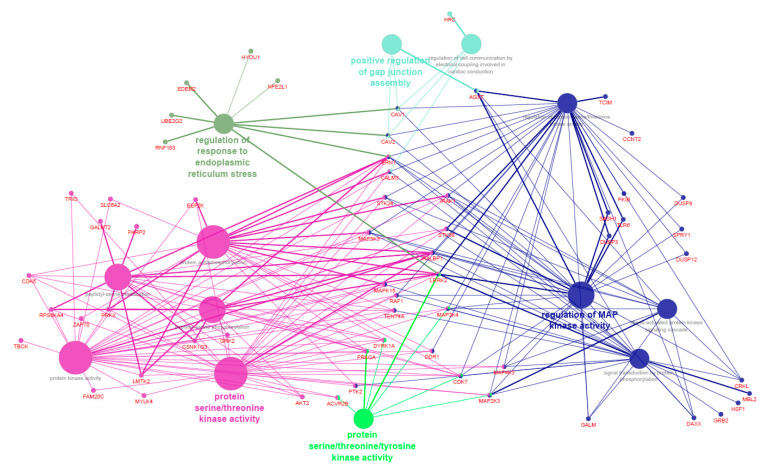
Functional enrichment analysis of the genes included in the modules significantly associated with oleic acid, *n*-6, and *n*-3 polyunsaturated fatty acids in pig *Semimembranosus* muscle. Different colors are used to highlight the significant Gene Ontology (GO) terms and the main genes entering in each GO term.

Supplementary File S5 reports the functional categories identified with DAVID for the genes significantly entering in the modules associated with oleic acid, *n*-6, and *n*-3 PUFAs (i.e., skyblue1, antiquewhite2, darkorange, darkseagreen3, darkgreen, and steelblue). Figure 3 shows the Cytoscape functional annotation enrichment for the same genes. These genes were significantly enriched in “GO:1905897 Regulation of response to endoplasmic reticulum stress”, “GO:0004712 Protein serine/threonine/tyrosine kinase activity”, “GO:1901844 Regulation of cell communication by electrical coupling involved in cardiac conduction”, “GO:1903598 Positive regulation of gap junction assembly”, “GO:0031098 Stress-activated protein kinase signaling cascade”, “GO:0023014 Signal transduction by protein phosphorylation”, “GO:0071900 Regulation of protein serine/threonine kinase activity”, “GO:0043405 Regulation of MAP kinase activity”, “GO:0004672 Protein kinase activity”, “GO:0018209 Peptidyl-serine modification”, “GO:0046777 Protein autophosphorylation”, “GO:0018105 Peptidyl-serine phosphorylation”, and “GO:0004674 Protein serine/threonine kinase activity” (all *p* < 0.001). On the whole, both functional analysis tools suggested that the genes included in skyblue1, antiquewhite2, darkorange, darkseagreen3, darkgreen, and steelblue modules were significantly enriched in pathways involved in serine/threonine kinase activity.

## 4. Discussion

The purpose of this study was to investigate the gene co-expression networks related to the palmitic, palmitoleic, stearic, oleic, *n*-6, and *n*-3 PUFA percentages in ILW pigs’ SM. This study starts from the results of Zappaterra et al. [13], where we investigated, in the same samples used in the present research, the differentially expressed genes and the gene networks associated with the divergent contents of SM IMF.

Palmitic, palmitoleic, and oleic acids showed trends of gene module-trait correlations similar to those observed for IMF content, suggesting that these traits may share, at least in part, a common genetic background. This evidence is consistent with our previous study [11], where positive genetic correlations were identified between these three FAs, and between these FAs and SM IMF content in ILW pigs. In agreement with these observations, oleic acid content was correlated with IMF deposition also in other porcine breeds and muscles. The content of oleic acid showed, indeed, positive genetic correlations with the IMF deposition in *Longissimus dorsi* [39], *Semimembranosus* [39], and *Gluteus medius* muscles of Duroc pigs [39,40]. Furthermore, the gene modules mostly associated with IMF and discussed in our previous study [13] also showed significant positive correlations with some FAs considered in the present study. In Zappaterra et al. [13], the gene networks considered for further discussion were those comprised in the modules skyblue1, darkturquoise, lavenderblush3, and grey60, which contained genes regulating DNA transcription and cell differentiation, primary cilia morphogenesis, ERK/MAPK, and G protein signaling cascades. In the current study, the module skyblue1 was associated with oleic and *n*-6 PUFAs, lavenderblush3 was significantly related to palmitic acid proportion, and grey60 showed positive module-trait correlations with palmitic and palmitoleic acid contents (whose value, however, did not exceed the threshold of |0.70|). This overlapping between the modules associated with IMF and the FAs considered in this study seems therefore to suggest that the same gene networks involved in IMF deposition may also play a role in the muscle metabolism of palmitic, palmitoleic, oleic, *n*-6, and *n*-3 PUFAs. In addition to those modules, the present study permitted the identification of new gene networks associated with the considered FAs, suggesting that FA composition variability relies only in part on the gene networks associated with IMF deposition.

On the whole, palmitic, palmitoleic, and oleic acids showed similar module-trait correlations when compared with IMF, while *n*-3 and *n*-6 PUFAs showed opposite trends in gene module-trait correlations when compared to those observed for IMF content. This result suggests that the gene networks linked to increased IMF deposition are associated with decreased depositions of *n*-3 and *n*-6 PUFAs. The negative relation between fat deposition and FA unsaturation degree is well known in the literature. PUFAs are indeed mainly incorporated in membrane phospholipids and play a role in membrane flexibility, inflammation control, eicosanoid production, plasma triacylglycerol synthesis, and gene expression regulation [41]. For these reasons, in individuals fed the same diet, PUFA absolute content remains quite stable in animal tissues over time and does not depend on fat deposition [42]. On the other side, increased fat deposition implies greater synthesis and storage of triacylglycerols, which are mainly constituted by SFAs and MUFAs, determining an increase in SFAs and MUFAs and a decreased proportion of PUFAs of the total FAs [42]. The trends in gene module-trait correlations observed in the present study are, therefore, in agreement with the knowledge concerning the FA synthesis and metabolism in animal tissues.

Intriguingly, unlike the above-mentioned FAs, stearic acid did not show modules with correlation values above the threshold of |0.70|. In animal tissues, stearic acid may either be obtained from the diet or synthesized by the elongation of palmitic acid. Even when compared with other SFAs, such as palmitic and myristic acids, stearic acid was found to be a less efficient substrate for triglyceride synthesis [43]. Its content in porcine subcutaneous tissue was found to increase between early life and slaughtering (at 90–110 kg live weight), suggesting that stearic acid deposition increases as subcutaneous fat becomes thicker [44]. When compared to subcutaneous fat, IMF is less dependent on diet and develops in a later stage of life [45,46], suggesting that stearic acid content may be less variable in muscle compared to subcutaneous fat depots. Furthermore, this FA is also the major substrate for the enzyme stearoyl-CoA desaturase (SCD), which catalyzes the conversion of stearic acid to oleic acid [43]. Thus, the fact that none of the observed gene modules obtained for stearic acid exceeded the defined threshold may depend on the role of this FA as an intermediate compound that undergoes several subsequent conversion steps through different metabolic pathways. The lack of strong correlations between stearic acid and the gene modules in the present study may, however, also be determined by the fact that stearic amounts in porcine tissues have smaller variation ranges when compared with other FAs [47].

Another objective of the current study was to find gene networks involved in the deposition of the considered FAs and identify possible genes of interest for the improvement of muscle FA composition. The percentage of palmitic and palmitoleic acids in IMF was associated with genes involved in intracellular distribution of mitochondria and mitochondrial activity, regulation of endothelial cell migration, positive regulation of autophagy, positive regulation of membrane attack complex, and cytosolic transport. The observed relation between the mitochondrial activity and the content of palmitic acid has already been reported in the literature. Palmitic acid is indeed a major FA used for energy supply, via adenosine triphosphate (ATP) production through FA oxidation in mitochondria. However, an excessive amount of palmitic acid was proven to cause lipid accumulation and induce endoplasmic reticulum stress and mitochondrial dysfunction in hepatic and pancreatic cells [48,49]. This evidence suggests that palmitic acid functions both as a substrate and as a regulator of mitochondrial activity. The literature reports that this FA is known to play a pivotal role in the occurrence of mitochondrial dysfunction, lipotoxicity, and cell death [50]. However, full agreement on the negative effect of this FA is lacking in the literature, as a study on rat liver mitochondria showed that palmitic acid may not cause cytotoxicity or cell death, and that these effects may be triggered by high amounts of palmitoleic acid [51]. In the present study, we found palmitic and palmitoleic acid contents associated with the same gene co-expression networks, suggesting that the deposition of these two FAs was associated with similar molecular pathways. The genes involved in mitochondrial activity and distribution were less expressed in the samples with the highest contents of palmitic and palmitoleic acids. In particular, the expression of *Clustered Mitochondria Homolog* (*CLUH*) and *Microtubule Associated Protein Tau* (*MAPT*) genes, reported in the literature as genes involved in mitochondrial activity and distribution, had strong and negative gene significance values with palmitic and palmitoleic acids. This result suggests that these genes were less expressed in pigs displaying higher SM contents of palmitic and palmitoleic acids, which may indicate a lower mitochondrial activity in those samples, possibly leading to an increased deposition of palmitic and palmitoleic acids and IMF. Though our hypothesis relies only on gene expression results, and further investigations are needed to confirm it, the role of mitochondrial function in the regulation of IMF deposition in pigs is well established [52]. However, as palmitic acid can be both a substrate and a regulator of mitochondrial activity, we are not able to discern whether the altered mitochondrial function is the leading cause of palmitic accumulation or whether the increased palmitic acid accumulation has led to a lower expression of genes related to mitochondrial function. The hypothesized altered mitochondrial function in muscle samples with high palmitic acid seems also to be supported by the downregulation of *Mitofusin 2* (*MFN2*) gene in the same samples. This gene encodes a mitochondrial membrane protein that is critical for mitochondrial fusion and mitochondrial clustering, and its downregulation has been found to cause mitochondrial dysfunction and altered Ca2+ homeostasis in neurons [53]. Interestingly, deficiencies in mitochondrial fusion have been found to disrupt the normal metabolic routes involved in FA oxidation, rerouting towards intracellular lipid droplets the FAs that should have been used in FA oxidation [54]. During starvation, normal FA trafficking and oxidation inside the cell may be mediated by two mechanisms: one requires autophagic digestion of lipid droplets; the other is by lipolytic consumption of lipid droplets [54]. The first involves autophagosomal engulfment of the lipid droplets and fusion with the lysosome, where hydrolytic enzymes digest the lipids stored in the lipid droplets, releasing free FAs into the cytoplasm that are then oxidized by mitochondria [55]. Intriguingly, in the present study, we found several genes involved in the autophagy regulation being downregulated in samples with high palmitic acid. In particular, in addition to *MFN2*, high palmitic and palmitoleic acid contents in SM were associated with a downregulation of the genes *La Ribonucleoprotein 1, Translational Regulator* (*LARP1*), *RNA Binding Motif Protein 14* (*RBM14*), and *Serine/Threonine Kinase 11* (*STK11*), which are all genes whose expression activates autophagy [56,57,58]. Also, the gene *Leucine Rich Repeat Kinase 2* (*LRRK2*), upregulated in the samples with high IMF, palmitic, palmitoleic, and oleic acids, has been implicated in the literature as a modulator of the autophagy process [59]. Autophagy is a complex process playing a role of paramount importance in cell survival. This process is indeed required for balancing sources of energy at critical times in cell development, eliminating misfolded or aggregated proteins (aggresomes), clearing damaged organelles (such as mitochondria), and removing intracellular pathogens [60]. Recent studies highlighted the important role of autophagy in the regulation of lipid metabolism in the liver [61], and our results seem to suggest that some sort of autophagy regulation on lipid metabolism may also exist in muscle. In the samples with high palmitic and palmitoleic acid percentages, the under-expression of genes involved in the autophagy process may suggest a downregulation of the autophagy processes in those samples. This cue may be consistent with a possible decrease in autophagosomal engulfment of lipid droplets in samples with a high percentage of palmitic and palmitoleic acids. It is, however, not possible to state with certainty whether the observed downregulation of the genes related to autophagy is actually associated with lipid droplets utilization, and the suggested hypotheses would need further verification. It is worth noting that the gene with the most negative gene significance for palmitic acid found in the present study is *TEA domain transcription factor 4* (*TEAD4*), a transcription factor preferentially expressed in skeletal muscle that regulates the expression of muscle-specific genes [62]. At present, this gene is poorly investigated in livestock species and has been only cited once in a recently published paper investigating gene expression networks involved in cattle feed efficiency [63]. Intriguingly, this gene is highly conserved in mammals and controls mitochondrial transcription: a loss of *TEAD4* impairs the recruitment of mitochondrial RNA polymerase to mitochondrial DNA (mtDNA), resulting in a reduced expression of mtDNA-encoded electron transport chain, with impaired oxidative energy metabolism in mammalian cells [64]. Based on our results and the evidence in the literature, it is possible to hypothesize that *TEAD4* downregulation noticed in samples with high palmitic acid deposition may be associated with a possible reduction in mitochondrial FA oxidation. Though these hypotheses need further investigation to be supported, *TEAD4* has been recently indicated as a hub gene involved in feed efficiency traits in Nelore cattle [63], thus suggesting that this gene may play a role of paramount importance in the regulation of energy metabolism in livestock species.

The modules associated with the IMF contents of oleic, *n*-6, and *n*-3 were extremely linked to Mitogen-Activated Protein Kinases (MAPKs). This result agrees with the literature, where MAPK-related pathways have often been related to FA synthesis. In neoplastic cell cultures, increased MAPK levels were related to an upregulation of Sterol Regulatory Element Binding Protein 1 (SREBP-1) transcription factor levels and of Fatty Acid Synthesis (FAS) enzyme, ultimately causing an increase in FA synthesis [65]. Among MAPK members, in the present study, *Mitogen-Activated Protein Kinase 15* (*MAPK15*, also known as *ERK7* or *ERK8*) gene was found to be under-expressed in samples with high oleic acid and overexpressed when samples had high percentages of *n*-6 and *n*-3 PUFAs. Remarkably, this kinase was found to act as a suppressor of adipogenesis in *Drosophila* [66], suggesting an anti-anabolic role for this kinase. Our result thus supports the evidence in the literature, as *MAPK15* was under-expressed in the samples with a higher amount of oleic acid, whose content in muscle is positively related to IMF [11]. Consistently, this gene was upregulated in the samples with high percentages of *n*-3 and *n*-6 PUFAs, whose proportion is higher in samples with low IMF deposition [11,42]. *MAPK15* has also a role of primary importance in primary cilium formation in human cells, and its depletion disrupts the organization of these cellular organelles [67]. This result seems to support the interpretations reported in our previous study, where we hypothesized a role for primary cilia in adipogenesis, and several genes related to primary cilia morphogenesis were found differentially expressed [13]. Together with *MAPK15*, the expression of the gene *Mitogen-Activated Protein Kinase Kinase 4* (*MAP2K4*) was also negatively related to oleic acid and positively correlated with PUFAs. This gene suppressed the levels of peroxisomal proliferator-activated receptor γ (PPARγ) [68], a transcription factor highly expressed in adipocytes [69]. PPARγ is known to be a master regulator of adipogenesis and thermogenesis and an active coordinator of lipid metabolism and insulin sensitivity [69]. Despite the fact that its expression is particularly high in subcutaneous and visceral fat, lower levels of this gene were also found in skeletal muscle, where its mRNA expression was higher in the *Longissimus dorsi* muscle of pigs with high IMF contents [70]. Taken together, these results support the hypothesis that MAPK plays a role in the control of fat, and consequently oleic acid, deposition in SM, suppressing adipogenesis and therefore increasing the proportion of *n*-6 and *n*-3 PUFAs in the total FAs. Among the genes negatively related to oleic acid content is also *Apolipoprotein A-5* (*APOA5*), which is widely studied in humans for its role in regulating plasma triglycerides [71]. Of great interest is also the recent identification of the relative *APOA5* protein as a major regulator of triglyceride storage in hepatocyte intracellular lipid droplets, suggesting for this gene a role in obesity and metabolic syndromes [72]. A higher expression of this protein was associated in humans with an impaired triglyceride storage [72] and a decreased uptake of FA in cardiomyocites [73]. Mutations in the porcine *APOA5* gene sequence were also found associated with meat quality traits in a Chinese pig breed characterized by high IMF deposition [74]. Taken together, these pieces of evidence suggest that this gene may also play a role in muscle intracellular lipid droplets and in triglyceride storage. The increased expression of the *APOA5* gene noticed in the samples with low oleic acid (and thus lower IMF contents) may indeed support the hypothesis that, in these samples, triglyceride utilization was promoted by *APOA5*, which activated lipid droplet mobilization. On the other hand, the gene whose expression correlated the most with oleic acid content was *WD Repeat Domain 19* (*WDR19, alias IFT144*). Its mRNA level was positively correlated with oleic acid and negatively related to PUFA proportions in SM, suggesting that this gene may be also involved in IMF deposition. Once again, this gene was involved in primary cilia morphology [75], emphasizing also in livestock species the possible role of primary cilium-related genes in fat deposition and FA composition. In agreement with this result, *Phosphatidylinositol-4-Phosphate 3-Kinase Catalytic Subunit Type 2* Alpha (*PIK3C2A*) gene was also found among the genes with the highest (and positive) gene significance for oleic acid content. A wide range of biological functions have been attributed to *PIK3C2A*, including its effects as a regulator of autophagy, glucose transport [76], and primary cilia formation and function [77]. The phosphoinositide 3-kinase-protein kinase B/AKT (PI3K-PKB/AKT) pathway is one of the most important molecular signaling pathways implicated in several cellular processes, including energy metabolism (i.e., glucose uptake), and in cell growth, proliferation, motility, and survival [78]. In our study, the samples overexpressing *PIK3C2A* showed also a higher expression of *Solute Carrier Family 2 Member 4* (*SLC2A4*, alias *GLUT4*). *SLC2A4* gene encodes a sugar transporter protein that catalyzes hexose transport across cellular membranes through an ATP-independent mechanism and is highly expressed in adipose tissue and skeletal muscle [79]. Its upregulation in the SM samples with a high content of oleic may thus be a direct reflection of the highest content of IMF in those animals, which is highly dependent on the number of muscle inter-dispersed adipocytes [80].

On the whole, the obtained results highlight the gene networks associated with FA variability in porcine SM. Transcriptomic analyses and weighted gene co-expression networks are important tools for disentangling the molecular scenario associated with phenotypic variability and suggest new genes of interest for performing further investigations. Because of the limited sample size, the results should be considered with caution and need further confirmation. Some of the identified molecular networks may indeed be biased by the number of samples, which may have determined a background noise and spuriously correlated gene pairs. Therefore, though the molecular patterns and key genes discussed in the present study seem to be consistent with the literature, further investigations are needed to validate the hypothesized molecular cascades associated with palmitic, palmitoleic, oleic, *n*-3, and *n*-6 PUFAs.

## 5. Conclusions

To the best of our knowledge, this is the first study investigating the gene expression networks associated with the palmitic, palmitoleic, stearic, oleic, *n*-6, and *n*-3 PUFA percentages in ILW pig SM. The obtained results confirmed the results of our previous study on IMF deposition, suggesting a possible involvement of genes regulating primary cilia morphology in muscle FA composition. SM samples with high percentages of palmitic and palmitoleic acids showed an under-expression of genes involved in autophagy, mitochondrial fusion, and mitochondrial activity. Among the key genes related to these FAs, we found *TEAD4*, a gene regulating mitochondrial activity, which seems to be a promising candidate for further studies aimed at finding the key molecular drivers involved in the control of cell energy metabolism. On the other hand, the genes comprised in the modules associated with the IMF contents of oleic, *n*-6, and *n*-3 PUFAs were significantly enriched in MAPK signaling, and several genes encoding for kinases (in particular, *MAPK15* and *MAP2K4*) may be important players regulating lipid homeostasis in muscle tissue. Except for *APOA5*, we found few genes encoding for enzymes with a direct activity on lipid substrates, suggesting that lipid metabolism and FA composition in muscle are mediated by complex intracellular signaling cascades regulating energy flux and intracellular lipid droplet utilization. The obtained results improve the knowledge of the gene networks involved in muscle FA composition, suggesting the possibility that, if confirmed, the discussed genes may be candidates to be included in selection schemes for pork quality.

## Figures and Tables

**Table 1:** Sex, hot carcass weight (kg), intramuscular fat (IMF) %, and *Semimembranosus* muscle (SM) fatty acid composition (%) of the 12 pigs selected for the transcriptome analysis of SM. PUFAs stands for polyunsaturated fatty acids.

Sample	Sex	Carcass Weight (kg)	IMF (%)	Palmitic Acid (%)	Palmitoleic Acid (%)	Stearic Acid (%)	Oleic Acid (%)	*n*-6 PUFAs (%)	*n*-3 PUFAs (%)
1	Gilt	132	8.64	22.94	2.88	10.81	37.63	18.68	0.88
2	Gilt	120	7.82	22.13	2.15	10.26	27.76	31.07	1.30
3	Gilt	105	6.65	23.63	3.10	11.56	40.06	13.78	0.72
4	Barrow	126	5.99	23.93	3.07	12.66	38.90	13.76	0.58
5	Barrow	120	5.89	24.33	2.34	13.30	42.80	10.15	0.49
6	Barrow	120	5.87	25.19	3.51	11.74	44.16	7.83	0.47
7	Barrow	120	0.74	24.25	3.13	12.95	44.94	7.72	0.45
8	Barrow	124	0.73	24.76	2.73	13.25	41.58	10.74	0.59
9	Gilt	119	0.71	24.04	3.03	11.69	40.81	13.28	0.72
10	Gilt	110	0.67	22.13	2.27	11.60	45.22	11.52	0.55
11	Barrow	127	0.64	22.89	2.59	13.72	36.50	16.94	0.77
12	Gilt	116	0.51	24.22	2.42	14.68	32.10	19.38	0.71

**Table 2:** List of the gene modules significantly correlated with the analyzed traits. Bold values indicate correlations ≥ |0.70|. PUFAs stands for polyunsaturated fatty acids.

Gene Modules	IMF (%)	Palmitic Acid (%)	Palmitoleic Acid (%)	Stearic Acid (%)	Oleic Acid (%)	*n*-6 PUFAs (%)	*n*-3 PUFAs (%)
MEskyblue1	0.65	0.66	0.67	0.08	**0.71**	**−0.76**	−0.66
MEgrey60	**0.77**	0.61	0.61	−0.09	0.51	−0.53	−0.45
MEantiquewhite2	0.15	0.58	0.36	0.19	**0.73**	**−0.77**	**−0.77**
MEyellowgreen	0.44	0.68	**0.71**	0.03	0.24	−0.37	−0.32
MEdarkorange	−0.25	−0.49	−0.43	−0.52	**−0.70**	**0.81**	**0.84**
MElavenderblush3	−0.63	**−0.80**	−0.60	−0.17	−0.22	0.39	0.35
MEdarkseagreen3	−0.55	−0.61	−0.30	−0.26	−0.65	**0.71**	**0.72**
MEdarkgreen	−0.21	−0.17	−0.37	−0.07	**−0.85**	**0.79**	**0.70**
MEsteelblue	−0.51	**−0.80**	−0.65	−0.16	−0.63	**0.73**	0.61

## Data Availability

The data presented in this study are openly available in the Gene Expression Omnibus (GEO) expression database under the accession number GSE144780.

## Supplementary Materials

The following are available online at https://www.mdpi.com/2076-2615/11/3/628/s1, Figure S1: Scale independence (on the right) and mean connectivity parameters (on the left) for the different values of Soft Threshold power. The highest R2 for the Scale Free Topology Model is indicated with the red line, and thus the first value of the Soft Threshold power located above the red line is the parameter chosen to obtain the best representation of the weighted gene co-expression network; Figure S2: Score plot of the Principal Component Analysis (PCA) performed on the normalized gene count matrix for the 12 samples of *Semimembranosus* muscle (SM). Different groups are indicated with different colors, and different shapes are used for the two sexes (F = females, M = barrows); File S1: The complete list of the module-trait relationships and the relative *p*-values for all identified gene modules and considered fatty acids. Module entries are arranged in alphabetical order; File S2: Gene Module Memberships (MM) and the relative *p*-values for all genes considered for the weighted gene co-expression analysis. Gene MM values indicate the correlation of the gene expression with the module eigengene and thus the importance of the gene in that module. Gene entries are arranged in alphabetical order; File S3: the list of the overall gene significance and the relative *p*-values of the genes on the considered fatty acids. The gene significance indicates the correlation between the gene expression and the trait; File S4: Results of DAVID functional enrichment analysis for the genes significantly entering in the gene modules associated with the percentages of palmitic and palmitoleic acids in the Italian Large White pig *Semimembranosus* muscle; File S5: Results of DAVID functional enrichment analysis for the genes significantly entering in the gene modules associated with the percentages of oleic acid, *n*-6 and *n*-3 polyunsaturated fatty acids in the Italian Large White pig *Semimembranosus* muscle.

## References

[1] OECD (2016). Meat. OECD-FAO Agricultural Outlook 2016-2025.

[2] Dalle Zotte A., Brugiapaglia A., Cullere M. (2017). What Is Meat in Italy?. Anim. Front..

[3] Ismea Mercati—Carni—Carne Suina e Salumi. http://www.ismeamercati.it/carni/carne-suina-salumi.

[4] Bosi P., Russo V. (2004). The Production of the Heavy Pig for High Quality Processed Products. Ital. J. Anim. Sci..

[5] Fiego D.P.L., Macchioni P., Minelli G., Santoro P. (2010). Lipid Composition of Covering and Intramuscular Fat in Pigs at Different Slaughter Age. Ital. J. Anim. Sci..

[6] Wood J.D., Enser M., Fisher A.V., Nute G.R., Sheard P.R., Richardson R.I., Hughes S.I., Whittington F.M. (2008). Fat Deposition, Fatty Acid Composition and Meat Quality: A Review. Meat Sci..

[7] Caggiula A.W., Mustad V.A. (1997). Effects of Dietary Fat and Fatty Acids on Coronary Artery Disease Risk and Total and Lipoprotein Cholesterol Concentrations: Epidemiologic Studies. Am. J. Clin. Nutr..

[8] Simopoulos A.P. (2008). The Importance of the Omega-6/Omega-3 Fatty Acid Ratio in Cardiovascular Disease and Other Chronic Diseases. Exp. Biol. Med. Maywood NJ.

[9] Lo Fiego D.P., Santoro P., Macchioni P., De Leonibus E. (2005). Influence of Genetic Type, Live Weight at Slaughter and Carcass Fatness on Fatty Acid Composition of Subcutaneous Adipose Tissue of Raw Ham in the Heavy Pig. Meat Sci..

[10] Kim Y.M., Choi T.J., Ho Cho K., Cho E.S., Lee J.J., Chung H.J., Baek S.Y., Jeong Y.D. (2018). Effects of Sex and Breed on Meat Quality and Sensory Properties in Three-Way Crossbred Pigs Sired by Duroc or by a Synthetic Breed Based on a Korean Native Breed. Korean J. Food Sci. Anim. Resour..

[11] Zappaterra M., Catillo G., Belmonte A.M., Lo Fiego D.P., Zambonelli P., Steri R., Buttazzoni L., Davoli R. (2020). Genetic Parameters of Muscle Fatty Acid Profile in a Purebred Large White Heavy Pig Population. Meat Sci..

[12] Catillo G., Zappaterra M., Zambonelli P., Buttazzoni L., Steri R., Minelli G., Davoli R. (2020). Genome-Wide Association Study Identifies Quantitative Trait Loci Regions Involved in Muscle Acidic Profile in Large White Heavy Pigs. Animal.

[13] Zappaterra M., Gioiosa S., Chillemi G., Zambonelli P., Davoli R. (2020). Muscle Transcriptome Analysis Identifies Genes Involved in Ciliogenesis and the Molecular Cascade Associated with Intramuscular Fat Content in Large White Heavy Pigs. PLoS ONE.

[14] Trapnell C., Roberts A., Goff L., Pertea G., Kim D., Kelley D.R., Pimentel H., Salzberg S.L., Rinn J.L., Pachter L. (2012). Differential Gene and Transcript Expression Analysis of RNA-Seq Experiments with TopHat and Cufflinks. Nat. Protoc..

[15] Puig-Oliveras A., Ramayo-Caldas Y., Corominas J., Estellé J., Pérez-Montarelo D., Hudson N.J., Casellas J., Folch J.M., Ballester M. (2014). Differences in Muscle Transcriptome among Pigs Phenotypically Extreme for Fatty Acid Composition. PLoS ONE.

[16] Zhang J., Cui L., Ma J., Chen C., Yang B., Huang L. (2017). Transcriptome Analyses Reveal Genes and Pathways Associated with Fatty Acid Composition Traits in Pigs. Anim. Genet..

[17] Piórkowska K., Żukowski K., Ropka-Molik K., Tyra M., Gurgul A., Piórkowska K., Żukowski K., Ropka-Molik K., Tyra M., Gurgul A. (2018). A Comprehensive Transcriptome Analysis of Skeletal Muscles in Two Polish Pig Breeds Differing in Fat and Meat Quality Traits. Genet. Mol. Biol..

[18] Schleinitz D., Krause K., Wohland T., Gebhardt C., Linder N., Stumvoll M., Blüher M., Bechmann I., Kovacs P., Gericke M. (2020). Identification of Distinct Transcriptome Signatures of Human Adipose Tissue from Fifteen Depots. Eur. J. Hum. Genet..

[19] Zambonelli P., Gaffo E., Zappaterra M., Bortoluzzi S., Davoli R. (2016). Transcriptional Profiling of Subcutaneous Adipose Tissue in Italian Large White Pigs Divergent for Backfat Thickness. Anim. Genet..

[20] Li M., Wu H., Wang T., Xia Y., Jin L., Jiang A., Zhu L., Chen L., Li R., Li X. (2012). Co-Methylated Genes in Different Adipose Depots of Pig Are Associated with Metabolic, Inflammatory and Immune Processes. Int. J. Biol. Sci..

[21] Borkman M., Storlien L.H., Pan D.A., Jenkins A.B., Chisholm D.J., Campbell L.V. (1993). The Relation between Insulin Sensitivity and the Fatty-Acid Composition of Skeletal-Muscle Phospholipids. N. Engl. J. Med..

[22] Garaulet M., Pérez-Llamas F., Pérez-Ayala M., Martínez P., de Medina F.S., Tebar F.J., Zamora S. (2001). Site-Specific Differences in the Fatty Acid Composition of Abdominal Adipose Tissue in an Obese Population from a Mediterranean Area: Relation with Dietary Fatty Acids, Plasma Lipid Profile, Serum Insulin, and Central Obesity. Am. J. Clin. Nutr..

[23] Garaulet M., Hernandez-Morante J.J., Lujan J., Tebar F.J., Zamora S. (2006). Relationship between Fat Cell Size and Number and Fatty Acid Composition in Adipose Tissue from Different Fat Depots in Overweight/Obese Humans. Int. J. Obes..

[24] Kriketos A.D., Pan D.A., Lillioja S., Cooney G.J., Baur L.A., Milner M.R., Sutton J.R., Jenkins A.B., Bogardus C., Storlien L.H. (1996). Interrelationships between Muscle Morphology, Insulin Action, and Adiposity. Am. J. Physiol..

[25] Davoli R., Luise D., Mingazzini V., Zambonelli P., Braglia S., Serra A., Russo V. (2016). Genome-Wide Study on Intramuscular Fat in Italian Large White Pig Breed Using the PorcineSNP60 BeadChip. J. Anim. Breed. Genet..

[26] Association of Official Analytical Chemists (AOAC) (2005). Official Methods of Analysis, Method 920.39, Fat (Crude) or Ether Extract in Animal Feed.

[27] Bolger A.M., Lohse M., Usadel B. (2014). Trimmomatic: A Flexible Trimmer for Illumina Sequence Data. Bioinforma. Oxf. Engl..

[28] Kim D., Langmead B., Salzberg S.L. (2015). HISAT: A Fast Spliced Aligner with Low Memory Requirements. Nat. Methods.

[29] Pertea M., Pertea G.M., Antonescu C.M., Chang T.-C., Mendell J.T., Salzberg S.L. (2015). StringTie Enables Improved Reconstruction of a Transcriptome from RNA-Seq Reads. Nat. Biotechnol..

[30] Anders S., Pyl P.T., Huber W. (2015). HTSeq--A Python Framework to Work with High-Throughput Sequencing Data. Bioinforma. Oxf. Engl..

[31] Love M.I., Huber W., Anders S. (2014). Moderated estimation of fold change and dispersion for RNA-seq data with DESeq2. Genome Biol..

[32] R Core Team (2020). R: A Language and Environment for Statistical Computing.

[33] Langfelder P., Horvath S. (2008). WGCNA: An R Package for Weighted Correlation Network Analysis. BMC Bioinform..

[34] Pampouille E., Hennequet-Antier C., Praud C., Juanchich A., Brionne A., Godet E., Bordeau T., Fagnoul F., Le Bihan-Duval E., Berri C. (2019). Differential Expression and Co-Expression Gene Network Analyses Reveal Molecular Mechanisms and Candidate Biomarkers Involved in Breast Muscle Myopathies in Chicken. Sci. Rep..

[35] Huang D.W., Sherman B.T., Lempicki R.A. (2009). Systematic and Integrative Analysis of Large Gene Lists Using DAVID Bioinformatics Resources. Nat. Protoc..

[36] Shannon P., Markiel A., Ozier O., Baliga N.S., Wang J.T., Ramage D., Amin N., Schwikowski B., Ideker T. (2003). Cytoscape: A Software Environment for Integrated Models of Biomolecular Interaction Networks. Genome Res..

[37] Bindea G., Mlecnik B., Hackl H., Charoentong P., Tosolini M., Kirilovsky A., Fridman W.-H., Pagès F., Trajanoski Z., Galon J. (2009). ClueGO: A Cytoscape Plug-in to Decipher Functionally Grouped Gene Ontology and Pathway Annotation Networks. Bioinformatics.

[38] Bindea G., Galon J., Mlecnik B. (2013). CluePedia Cytoscape Plugin: Pathway Insights Using Integrated Experimental and in Silico Data. Bioinforma. Oxf. Engl..

[39] Ros-Freixedes R., Reixach J., Bosch L., Tor M., Estany J. (2014). Genetic Correlations of Intramuscular Fat Content and Fatty Acid Composition among Muscles and with Subcutaneous Fat in Duroc Pigs1. J. Anim. Sci..

[40] Ros-Freixedes R., Reixach J., Tor M., Estany J. (2012). Expected Genetic Response for Oleic Acid Content in Pork1. J. Anim. Sci..

[41] Fernandez M.L., West K.L. (2005). Mechanisms by Which Dietary Fatty Acids Modulate Plasma Lipids. J. Nutr..

[42] De Smet S., Raes K., Demeyer D. (2004). Meat Fatty Acid Composition as Affected by Fatness and Genetic Factors: A Review. Anim. Res..

[43] Sampath H., Ntambi J.M. (2005). The Fate and Intermediary Metabolism of Stearic Acid. Lipids.

[44] Wood J.D., Wiseman J. (1984). 20—Fat deposition and the quality of fat tissue in meat animals. Fats in Animal Nutrition.

[45] Hausman G.J., Dodson M.V., Ajuwon K., Azain M., Barnes K.M., Guan L.L., Jiang Z., Poulos S.P., Sainz R.D., Smith S. (2009). BOARD-INVITED REVIEW: The Biology and Regulation of Preadipocytes and Adipocytes in Meat Animals1,2. J. Anim. Sci..

[46] Hamill R.M., Aslan O., Mullen A.M., O’Doherty J.V., McBryan J., Morris D.G., Sweeney T. (2013). Transcriptome Analysis of Porcine M. Semimembranosus Divergent in Intramuscular Fat as a Consequence of Dietary Protein Restriction. BMC Genom..

[47] Wood J.D., Richardson R.I., Nute G.R., Fisher A.V., Campo M.M., Kasapidou E., Sheard P.R., Enser M. (2004). Effects of Fatty Acids on Meat Quality: A Review. Meat Sci..

[48] Jun D.W., Cho W.K., Jun J.H., Kwon H.J., Jang K.-S., Kim H.-J., Jeon H.J., Lee K.N., Lee H.L., Lee O.Y. (2011). Prevention of Free Fatty Acid-Induced Hepatic Lipotoxicity by Carnitine via Reversal of Mitochondrial Dysfunction. Liver Int. Off. J. Int. Assoc. Study Liver.

[49] Hirata T., Kawai T., Hirose H., Tanaka K., Kurosawa H., Fujii C., Fujita H., Seto Y., Matsumoto H., Itoh H. (2016). Palmitic Acid-Rich Diet Suppresses Glucose-Stimulated Insulin Secretion (GSIS) and Induces Endoplasmic Reticulum (ER) Stress in Pancreatic Islets in Mice. Endocr. Res..

[50] Sparagna G.C., Hickson-Bick D.L., Buja L.M., McMillin J.B. (2001). Fatty Acid-Induced Apoptosis in Neonatal Cardiomyocytes: Redox Signaling. Antioxid. Redox Signal..

[51] Penzo D., Tagliapietra C., Colonna R., Petronilli V., Bernardi P. (2002). Effects of Fatty Acids on Mitochondria: Implications for Cell Death. Biochim. Biophys. Acta BBA-Bioenerg..

[52] Liu K., Yu W., Wei W., Zhang X., Tian Y., Sherif M., Liu X., Dong C., Wu W., Zhang L. (2019). Melatonin Reduces Intramuscular Fat Deposition by Promoting Lipolysis and Increasing Mitochondrial Function. J. Lipid Res..

[53] Martorell-Riera A., Segarra-Mondejar M., Muñoz J.P., Ginet V., Olloquequi J., Pérez-Clausell J., Palacín M., Reina M., Puyal J., Zorzano A. (2014). Mfn2 Downregulation in Excitotoxicity Causes Mitochondrial Dysfunction and Delayed Neuronal Death. EMBO J..

[54] Rambold A.S., Cohen S., Lippincott-Schwartz J. (2015). Fatty Acid Trafficking in Starved Cells: Regulation by Lipid Droplet Lipolysis, Autophagy, and Mitochondrial Fusion Dynamics. Dev. Cell.

[55] Singh R., Kaushik S., Wang Y., Xiang Y., Novak I., Komatsu M., Tanaka K., Cuervo A.M., Czaja M.J. (2009). Autophagy Regulates Lipid Metabolism. Nature.

[56] Muniraj N., Siddharth S., Shriver M., Nagalingam A., Parida S., Woo J., Elsey J., Gabrielson K., Gabrielson E., Arbiser J.L. (2020). Induction of STK11-Dependent Cytoprotective Autophagy in Breast Cancer Cells upon Honokiol Treatment. Cell Death Discov..

[57] Fonseca B.D., Zakaria C., Jia J.-J., Graber T.E., Svitkin Y., Tahmasebi S., Healy D., Hoang H.-D., Jensen J.M., Diao I.T. (2015). La-Related Protein 1 (LARP1) Represses Terminal Oligopyrimidine (TOP) MRNA Translation Downstream of MTOR Complex 1 (MTORC1). J. Biol. Chem..

[58] Jung C.H., Jun C.B., Ro S.-H., Kim Y.-M., Otto N.M., Cao J., Kundu M., Kim D.-H. (2009). ULK-Atg13-FIP200 Complexes Mediate MTOR Signaling to the Autophagy Machinery. Mol. Biol. Cell.

[59] Gómez-Suaga P., Hilfiker S. (2012). LRRK2 as a Modulator of Lysosomal Calcium Homeostasis with Downstream Effects on Autophagy. Autophagy.

[60] Glick D., Barth S., Macleod K.F. (2010). Autophagy: Cellular and Molecular Mechanisms. J. Pathol..

[61] Saito T., Kuma A., Sugiura Y., Ichimura Y., Obata M., Kitamura H., Okuda S., Lee H.-C., Ikeda K., Kanegae Y. (2019). Autophagy Regulates Lipid Metabolism through Selective Turnover of NCoR1. Nat. Commun..

[62] Benhaddou A., Keime C., Ye T., Morlon A., Michel I., Jost B., Mengus G., Davidson I. (2012). Transcription Factor TEAD4 Regulates Expression of Myogenin and the Unfolded Protein Response Genes during C2C12 Cell Differentiation. Cell Death Differ..

[63] De Lima A.O., Koltes J.E., Diniz W.J.S., de Oliveira P.S.N., Cesar A.S.M., Tizioto P.C., Afonso J., de Souza M.M., Petrini J., Rocha M.I.P. (2020). Potential Biomarkers for Feed Efficiency-Related Traits in Nelore Cattle Identified by Co-Expression Network and Integrative Genomics Analyses. Front. Genet..

[64] Kumar R.P., Ray S., Home P., Saha B., Bhattacharya B., Wilkins H.M., Chavan H., Ganguly A., Milano-Foster J., Paul A. (2018). Regulation of Energy Metabolism during Early Mammalian Development: TEAD4 Controls Mitochondrial Transcription. Development.

[65] Yang Y.-A., Han W.F., Morin P.J., Chrest F.J., Pizer E.S. (2002). Activation of Fatty Acid Synthesis during Neoplastic Transformation: Role of Mitogen-Activated Protein Kinase and Phosphatidylinositol 3-Kinase. Exp. Cell Res..

[66] Hasygar K., Deniz O., Liu Y., Gullmets J., Hynynen R., Ruhanen H., Kokki K., Käkelä R., Hietakangas V. (2021). Coordinated Control of Adiposity and Growth by Anti-Anabolic Kinase ERK7. EMBO Rep..

[67] Kazatskaya A., Kuhns S., Lambacher N.J., Kennedy J.E., Brear A.G., McManus G.J., Sengupta P., Blacque O.E. (2017). Primary Cilium Formation and Ciliary Protein Trafficking Is Regulated by the Atypical MAP Kinase MAPK15 in Caenorhabditis Elegans and Human Cells. Genetics.

[68] Ahn Y.-H., Yang Y., Gibbons D.L., Creighton C.J., Yang F., Wistuba I.I., Lin W., Thilaganathan N., Alvarez C.A., Roybal J. (2011). Map2k4 Functions as a Tumor Suppressor in Lung Adenocarcinoma and Inhibits Tumor Cell Invasion by Decreasing Peroxisome Proliferator-Activated Receptor γ2 Expression. Mol. Cell. Biol..

[69] Ma X., Wang D., Zhao W., Xu L. (2018). Deciphering the Roles of PPARγ in Adipocytes via Dynamic Change of Transcription Complex. Front. Endocrinol..

[70] Cui J., Chen W., Liu J., Xu T., Zeng Y. (2016). Study on Quantitative Expression of PPARγ and ADRP in Muscle and Its Association with Intramuscular Fat Deposition of Pig. SpringerPlus.

[71] Pennacchio L.A., Olivier M., Hubacek J.A., Cohen J.C., Cox D.R., Fruchart J.-C., Krauss R.M., Rubin E.M. (2001). An Apolipoprotein Influencing Triglycerides in Humans and Mice Revealed by Comparative Sequencing. Science.

[72] Su X., Kong Y., Peng D. (2018). New Insights into Apolipoprotein A5 in Controlling Lipoprotein Metabolism in Obesity and the Metabolic Syndrome Patients. Lipids Health Dis..

[73] Luo J., Xu L., Li J., Zhao S. (2018). Effects and Mechanisms of Apolipoprotein A-V on the Regulation of Lipid Accumulation in Cardiomyocytes. Lipids Health Dis..

[74] Hui Y.T., Yang Y.Q., Liu R.Y., Zhang Y.Y., Xiang C.J., Liu Z.Z., Ding Y.H., Zhang Y.L., Wang B.R. (2013). Significant Association of APOA5 and APOC3 Gene Polymorphisms with Meat Quality Traits in Kele Pigs. Genet. Mol. Res..

[75] Mariman E.C.M., Vink R.G., Roumans N.J.T., Bouwman F.G., Stumpel C.T.R.M., Aller E.E.J.G., van Baak M.A., Wang P. (2016). The Cilium: A Cellular Antenna with an Influence on Obesity Risk. Br. J. Nutr..

[76] Leibiger B., Moede T., Uhles S., Barker C.J., Creveaux M., Domin J., Berggren P.-O., Leibiger I.B. (2010). Insulin-Feedback via PI3K-C2α Activated PKBα/Akt1 Is Required for Glucose-Stimulated Insulin Secretion. FASEB J..

[77] Franco I., Gulluni F., Campa C.C., Costa C., Margaria J.P., Ciraolo E., Martini M., Monteyne D., De Luca E., Germena G. (2014). PI3K Class II α Controls Spatially Restricted Endosomal PtdIns3P and Rab11 Activation to Promote Primary Cilium Function. Dev. Cell.

[78] Nozhat Z., Hedayati M. (2016). PI3K/AKT Pathway and Its Mediators in Thyroid Carcinomas. Mol. Diagn. Ther..

[79] Huang S., Czech M.P. (2007). The GLUT4 Glucose Transporter. Cell Metab..

[80] Komolka K., Albrecht E., Wimmers K., Michal J.J., Maak S. (2014). Molecular Heterogeneities of Adipose Depots - Potential Effects on Adipose-Muscle Cross-Talk in Humans, Mice and Farm Animals. J. Genom..

