# Supplementary material for: Dissecting the Gene Expression Networks Associated with Variations in the Major Components of the Fatty Acid Semimembranosus Muscle Profile in Large White Heavy Pigs
Source: Animals (Basel). 2021 Feb 27;11(3):628. doi: 10.3390/ani11030628 (PMC7997476; doi:10.3390/ani11030628)
Supplement: Supplementary file 1 [file animals-11-00628-s001.zip › Supplementary material revised/Supplementary Figure S1 and Figure S2.docx]

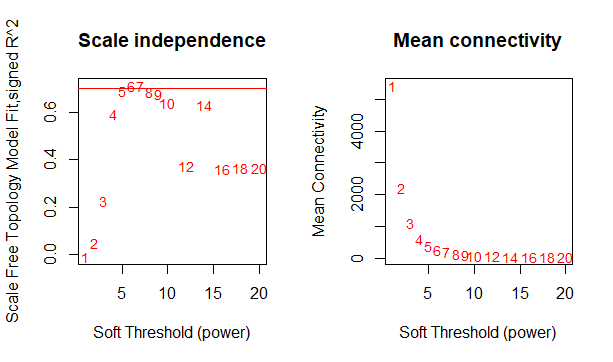


**Supplementary Figure S1.** Scale independence (on the right) and mean connectivity parameters (on the left) for the different values of Soft Threshold power. The highest R^2^ for the Scale Free Topology Model is indicated with the red line, and thus the first value of the Soft Threshold power located above the red line is the parameter chosen to obtain the best representation of the weighted gene co-expression network.


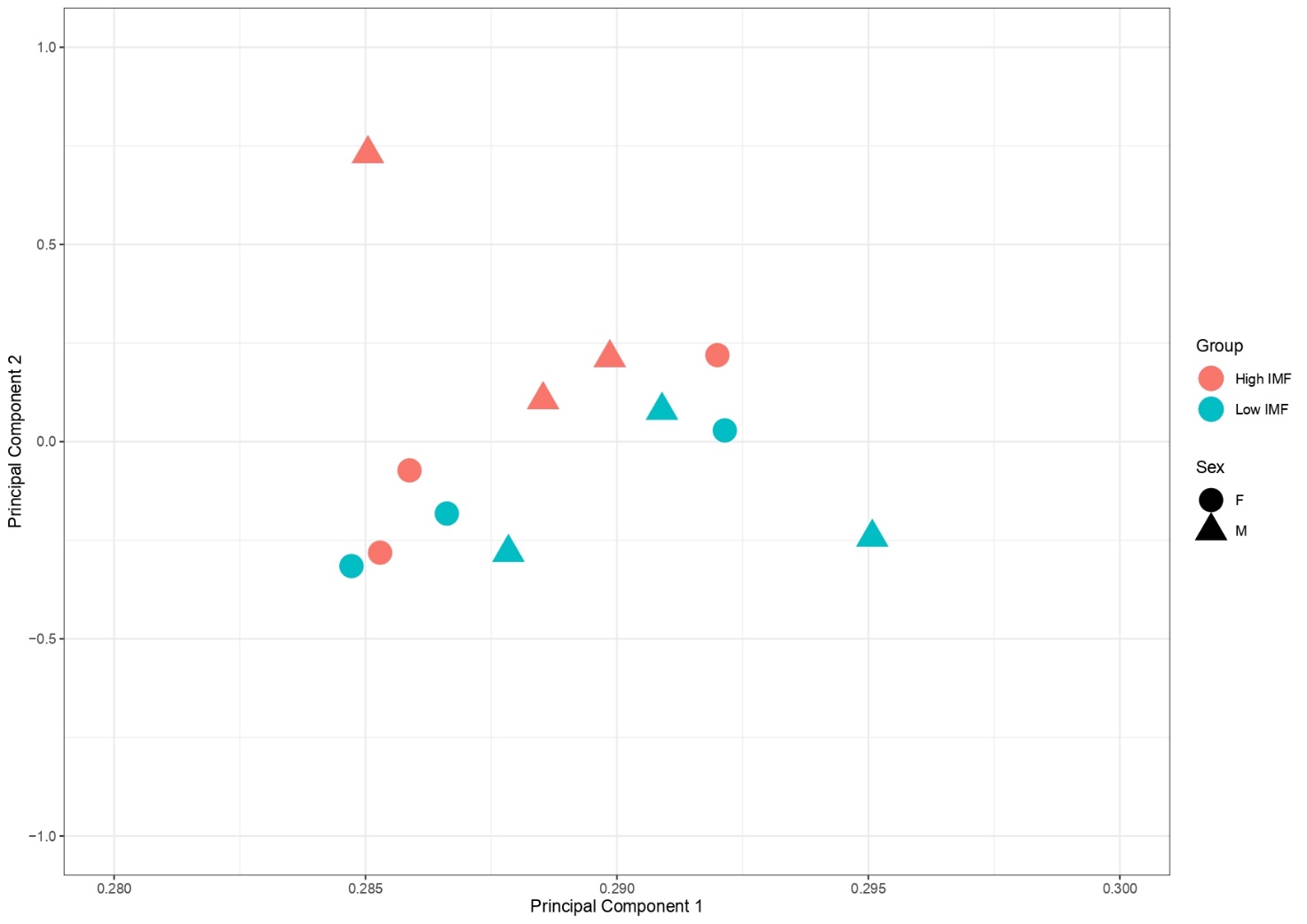


**Supplementary Figure S2.** Score plot of the Principal Component Analysis (PCA) performed on the normalized gene count matrix for the 12 samples of *Semimembranosus* muscle (SM). Different groups are indicated with different colors, and different shapes are used for the two sexes (F = females, M = barrows).
